# Provider advice on physical activity and nutrition in twin pregnancies: a cross-sectional electronic survey

**DOI:** 10.1186/s12884-019-2574-2

**Published:** 2019-11-14

**Authors:** Kara M. Whitaker, Meghan Baruth, Rebecca A. Schlaff, Hailee Talbot, Christopher P. Connolly, Jihong Liu, Sara Wilcox

**Affiliations:** 1 Department of Health and Human Physiology, University of Iowa, Iowa City, IA USA; 2 Department of Epidemiology, University of Iowa, Iowa City, IA USA; 3 Department of Health Sciences, Saginaw Valley State University, University Center, MI USA; 4 Department of Kinesiology, Saginaw Valley State University, University Center, MI USA; 5 Department of Kinesiology and Educational Psychology, Washington State University, Pullman, WA USA; 6 Department of Epidemiology and Biostatistics, University of South Carolina, Columbia, SC USA; 7 Department of Exercise Science, University of South Carolina, Columbia, SC USA

**Keywords:** Pregnancy, Twins, Physical activity, Nutrition, Health care provider, Advice, Counseling, Communication

## Abstract

**Background:**

Health care providers should counsel pregnant patients on physical activity and nutrition to improve pregnancy outcomes. However, little is known about provider advice on these lifestyle behaviors among women pregnant with twins, a growing population at high risk for pregnancy complications. We examined the prevalence and content of provider advice on physical activity and nutrition among women pregnant with twins.

**Methods:**

A cross-sectional electronic survey was administered to 276 women who delivered twins in the past 3 years and received prenatal care in the United States. The proportion of women reporting provider advice on physical activity and nutrition during prenatal visits (yes/no) was assessed and open-ended questions examined the content of provider advice. Bivariate differences in participant characteristics, stratified by provider advice on physical activity and nutrition (yes/no), were assessed. Responses from open-ended questions were examined using a content analysis approach to identify commonly reported advice on physical activity and nutrition.

**Results:**

Approximately 75 and 63% of women reported provider advice on physical activity and nutrition, respectively, during their twin pregnancy. Women who recalled advice on physical activity most commonly reported recommendations to walk at a light to moderate intensity level. However, few women reported physical activity recommendations consistent with current guidelines, and approximately 55% of women reported provider advice to limit or restrict activity during their pregnancy, including bedrest. Nutrition advice was focused on eating a healthy, balanced diet and increasing protein intake. More women reported self-initiating the conversation on physical activity with their provider (40%) compared to nutrition (21%). Despite limited advice, 70% of women reported being satisfied or very satisfied with the information they received from their provider on physical activity or nutrition.

**Conclusions:**

The majority of women reported provider advice on physical activity and nutrition during their twin pregnancies. However, advice was limited in detail, and physical activity levels were commonly restricted, despite the lack of evidence that activity restriction is beneficial during pregnancy. More research is needed to determine the optimal physical activity and dietary patterns in twin pregnancies to facilitate clear and consistent provider counseling on these lifestyle behaviors.

## Background

The twin birth rate has risen nearly 80% over the last four decades in the United States, accounting for 1 in every 30 births in 2016 [1]. Compared to singleton pregnancies, women pregnant with twins are at greater risk for adverse pregnancy outcomes, including hypertensive disorders, gestational diabetes, anemia, postpartum hemorrhage, operative delivery, uterine rupture, and prolonged hospitalization [2, 3]. Twin pregnancies are also associated with a 4- to 10-fold increased risk of perinatal morbidity and mortality compared to singleton pregnancies, largely driven by the increased risk of preterm birth, low birth weight, and intrauterine growth restriction [2, 3]. There are many non-modifiable risk factors that account for the disproportionate morbidity experienced in twin gestations. However, appropriate physical activity and proper nutrition during pregnancy are increasingly recognized as important modifiable factors that contribute to maternal and child outcomes [4].

Physical activity in pregnancy is associated with minimal risks and has been shown to provide health benefits to most women. The American College of Obstetricians and Gynecologists (ACOG) recommends all women with uncomplicated pregnancies engage in 20–30 min of moderate-intensity aerobic physical activity on most or all days of the week [4]. These recommendations are consistent with the U.S. Department of Health and Human Services (HHS) Physical Activity Guidelines for Americans, which state that women should do at least 150 min of moderate-intensity aerobic activity a week during pregnancy [5]. Health care providers who see pregnant women (e.g., obstetricians, midwives, nurse practitioners) are advised to carefully evaluate women with medical or obstetric complications before making recommendations on physical activity participation. There are no specific physical activity guidelines for women pregnant with twins; however, given the higher risk for maternal complications in twin gestations, it is important that health care providers evaluate the risks and benefits of physical activity for each patient and counsel accordingly.

Similarly, there are no specific nutritional guidelines for women pregnant with twins. Evidence suggests that compared to singleton pregnancies, maternal resting energy expenditure is approximately 10% higher in twin pregnancies [6]. This difference in resting energy expenditure could result in a 40% increase in caloric requirements [7]. Luke and colleagues suggest a daily caloric intake for twin pregnancies of 3000–3500 kcal/day for women of normal weight, 3250 kcal/day for overweight women, and 2700–3000 kcal/day for obese women, with 20% of energy intake derived from protein, 40% from low-glycemic index carbohydrates, and 40% from fat [8, 9]. Adequate protein intake is emphasized as essential to normal fetal growth in twin gestations. Iron, folate, calcium, magnesium, and zinc supplementation are also recommended beyond a usual prenatal vitamin [7]. Given the unique physiological demands placed on the body during a twin pregnancy, it is critical that health care providers counsel women on the importance of adequate caloric intake and provide guidance on macronutrient and micronutrient intake for optimal pregnancy outcomes.

There is growing evidence that health care provider advice on lifestyle behaviors is associated with women’s weight gain, physical activity, and dietary behaviors during singleton pregnancies [10–13]. However, little is known regarding whether or not health care providers discuss physical activity and nutrition with their patients who are pregnant with twins, or the content of physical activity and nutrition advice. This is especially important to evaluate given the limited physical activity and nutrition training reported in U.S. medical schools [14, 15]. The objectives of the current study were to: (1) determine the proportion of women who report health care provider advice on physical activity and nutrition during prenatal visits among women pregnant with twins, (2) examine whether participant characteristics are associated with health care provider advice on physical activity and nutrition, and (3) describe the content of health care provider advice on physical activity and nutrition in this population.

## Methods

### Study population

Participants in the Mothers of Twins Health Study were recruited through social media sites for mothers of multiples in May, 2018. Advertisements placed on websites (e.g., La Leche League for Mothers of Multiples) included a brief description of the survey with a link to access the screening form. Inclusion criteria were: twin birth within the last 3 years, first prenatal visit before 16 weeks gestation, knowledge of twin gestation prior to the third trimester, received prenatal care in the United States, 18–44 years of age, and not currently pregnant. Eligible participants were invited to complete a 15–20 min electronic survey to assess health behaviors and health care provider advice on weight gain, physical activity, and nutrition during their twin pregnancy. This paper focuses on findings related to physical activity and nutrition. Women who completed the full study survey received a $10 Amazon gift card. Informed consent was obtained from all participants and study protocols were approved by the University of Iowa Institutional Review Board. The datasets used and analyzed during the current study are available in the University of Iowa’s Institutional Repository [10.25820/mj2q-gj21].

### Provider advice on physical activity and nutrition

Women were asked if a health care provider (e.g., doctor, midwife, nurse) discussed physical activity with them during their twin pregnancy (yes, no, not sure). Women who responded affirmatively were subsequently asked in separate questions if a health care provider discussed: (1) types of physical activities they could participate in; (2) the intensity level or how hard they should be working while being physically active; (3) the amount of physical activity they should be getting (e.g., frequency and duration); (4) if physical activity advice changed as their pregnancy progressed; and (5) any other advice related to physical activity that was not already asked about (yes, no, not sure). If participants confirmed their provider discussed a given topic with them, they were asked what specific information they received from their provider using an open-ended response. It was therefore possible for women to respond to up to five open-ended questions on the content of physical activity advice (type, intensity, amount, change, and other). Participants were also asked to identify who started the conversation about physical activity (me, health care provider, not sure), and to select from a list which health care provider(s) had discussed physical activity during their twin pregnancy (ob/gyn, family practitioner, maternal-fetal medicine specialist, infertility specialist, midwife, nurse practitioner, nurse, dietician, other). Finally, women were asked how satisfied they were with the information they received from their healthcare provider on physical activity during their twin pregnancy, on a 5-point Likert scale from very dissatisfied to very satisfied. The questions on who started the conversation, which providers discussed physical activity, and satisfaction were asked for general physical activity advice, and not specifically for each physical activity content area.

Similarly, women were asked if a health care provider (e.g., doctor, midwife, nurse) discussed nutrition or healthy eating with them during their twin pregnancy (yes, no, not sure). Women who responded affirmatively were subsequently asked in an open-ended response what specific information they received from their provider on nutrition. Additionally, those who reported provider discussions on nutrition were also asked if nutrition advice changed as their pregnancy progressed and if a health care provider discussed how many calories they should be eating during their twin pregnancy (yes, no, not sure). Individuals who responded affirmatively were asked in separate questions to describe how advice changed or what recommendations were given related to caloric intake using open-ended responses. To reduce participant burden, we chose not to ask specific questions about various nutrition content areas due to the complexity of nutrition-related advice (e.g., carbohydrate, fat, and protein recommendations, macronutrient composition, fruits and vegetable consumption, specific recommended diet types, micronutrients, etc.). As done for physical activity, participants were asked who started the conversation about nutrition, which health care provider discussed the topic with them, and how satisfied they were with the advice received from their health care provider on nutrition during their twin pregnancy.

### Personal history questionnaire

Physical activity before and during pregnancy was assessed using the 2009 Behavioral Risk Factor Surveillance System (BRFSS) physical activity questionnaire [16]. This questionnaire has previously shown to provide similar group estimates for time spent in moderate and vigorous intensity physical activities compared to simultaneous heart-rate motions sensor techniques [17], and has demonstrated predictive validity with adverse health outcomes such as obesity [18, 19]. Participants were asked if they did any moderate or vigorous activities for at least 10 min at a time before they became pregnant with their twins. If women responded yes, they were then asked how many days per week they did these activities (separate questions for moderate and vigorous activities) for at least 10 min at a time (0–7), and how many total minutes per day they spent doing these activities (0, 10–20, 20–30, 30–40, 40–50, 50–60, and > 60). Zero response options were included for days per week and minutes per day as participants could report moderate, but not vigorous activities, or vice versa. This set of questions was repeated for the first, second and third trimesters of pregnancy. For the present study, modifications made to the original BRFSS physical activity questionnaire included repeating the questionnaire for pre-pregnancy and in each trimester of pregnancy while the original survey was only administered once, and we also provided a range of response options rather than using an open-ended response option for total minutes per day spent in moderate or vigorous activities. Average minutes per day of moderate to vigorous intensity physical activity (MVPA) were calculated by multiplying the number of days per week by the midpoint value within the selected interval (value of 65 used if > 60 was selected; < 10% of study population) before pregnancy, during each trimester, and averaged across trimesters.

Self-reported diet quality was assessed through a single-item measure that has been previously validated against the Healthy Eating Index-2010 [20]. Participants were asked: “In general, how healthy is your overall diet?” Response options were poor, fair, good, very good, or excellent, with a possible score range of 1 to 5, with higher scores indicating better diet quality. Before women answered this question they read the following description: “a healthy diet includes plenty of fruits and vegetables, low fat dairy products, protein, fiber, and whole grains (like whole wheat breads and brown rice) instead of refined grains (like white breads and race). A healthy diet includes watching portion sizes and avoiding eating too much of very sugary and fatty foods and drinks.” This question was used to assess diet before pregnancy as well as in the first, second, and third trimesters; scores across pregnancy trimesters were averaged.

Height and pre-pregnancy weight were ascertained by self-report and used to calculate pre-pregnancy body mass index (BMI; kg/m^2^). Pre-pregnancy BMI was categorized as underweight/normal weight (< 25.0 kg/m^2^), overweight (25.0–29.9 kg/m^2^), or obese (≥30.0 kg/m^2^). Additional measures included: maternal age at twin delivery, time since delivery in months, race, marital status, education, employment status, household income, parity prior to the twin pregnancy, use of assisted reproductive technologies for their twin pregnancy (yes/no), smoking status and alcohol consumption during the twin pregnancy, twin pregnancy type (dichorionic/diamniotic, dichorionic/monoamniotic, monochorionic/monoamniotic), pregnancy complications (gestational diabetes, high blood pressure, hypertension, preeclampsia, anemia, twin to twin transfusion syndrome, and hyperemesis gravidarum), gestational age at delivery, gestational weight gain, and twin A and B birthweights (very low birth weight (< 1500 g), low birth weight (1500–2499 g), normal birth weight (2500–4000 g), or high birth weight (> 4000 g)).

### Statistical analysis

Descriptive analyses, including frequencies and means, for key variables were conducted. Independent samples t-tests, Wilcoxon Mann Whitney tests, chi-square tests, or fisher’s exact tests were used to examine whether provider advice on physical activity (yes/no) and provider advice on nutrition (yes/no) differed by participant characteristics. In exploratory analyses, Wilcoxon Mann Whitney tests were used to examine associations between who initiated the conversation on physical activity (self vs. provider) with pre-pregnancy and pregnancy MVPA, as well as satisfaction with the physical activity advice received (categorized as dissatisfied, neutral, or satisfied). This process was repeated for nutrition advice, diet quality, and satisfaction with nutrition advice received.

Responses from open-ended questions assessing women’s report of provider advice on physical activity and diet were analyzed using NVivo 12 for computer-assisted qualitative data management. A content analysis approach was used to identify common recommendations for physical activity and nutrition [21]. Two authors (KW and HT) independently read and coded the data. Discussion and consensus between the two raters guided the organization of responses and frequencies and percentages were calculated.

## Results

**Fig. 1:**
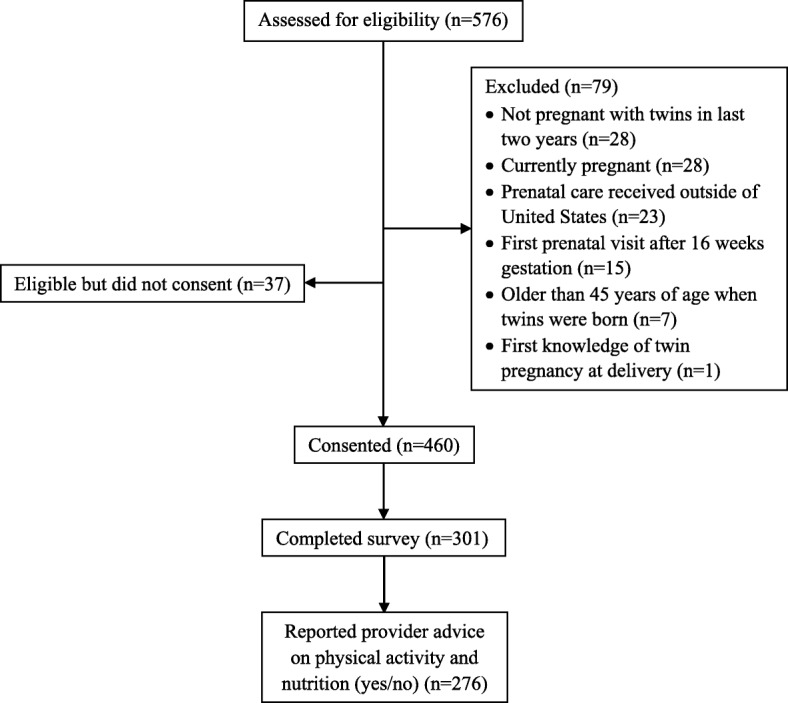
Mothers of twins health study participant flow chart

As seen in Fig. 1, 576 women were assessed for eligibility. Seventy-nine women were excluded for not meeting the eligibility criteria, and 37 women who met inclusion criteria did not consent to take part in the study. A total of 460 participants consented and began the survey, with 301 completing the full study survey. Of these, 276 women had complete data on health care provider advice on physical activity and nutrition (yes/no) and were included in analyses (52% of those screened for eligibility and 60% of those who consented). Note that 25 of the 301 women who completed the survey responded ‘not sure’ to provider advice on physical activity and nutrition and were excluded from analyses.*.*

Participant characteristics, overall and stratified by women’s report of provider advice on physical activity and nutrition, are shown in Table 1. Women averaged 31.4 ± 4.2 years of age and were 11.3 ± 7.7 months postpartum. The majority of participants were white, married, and over 80% had a college degree. Women who had children prior to the twin pregnancy were less likely to report provider advice on physical activity and nutrition during their twin pregnancy (*p* < 0.05). Women who reported provider advice on physical activity were more likely to be underweight/normal weight and less likely to be overweight, had more minutes/day of MVPA during the first and second trimesters, and delivered their babies earlier compared to those who did not report provider advice on physical activity (all *p* < 0.05). Women who reported provider advice on nutrition had higher diet quality scores in the first and third trimesters, and were more likely to have very low or low birth weight babies (all p < 0.05). Report of provider advice on physical activity or nutrition was unrelated to maternal age, time since delivery, race, marital status, education, employment, income, use of assisted reproductive technologies, smoking, alcohol use, twin pregnancy type, pregnancy complications, or adequacy of gestational weight gain.

**Table 1:** Participant characteristics, overall and by provider advice on physical activity (PA) and nutrition, *N* = 276

Participant Characteristics	Total *N* = 276	Advised on PA *N* = 208	Not Advised on PA *N* = 68	*p*-value^a^	Advised on Nutrition *N* = 173	Not Advised on Nutrition *N* = 103	*p*-value^a^
Age at delivery, mean years ± SD	31.4 ± 4.2	31.3 ± 4.3	31.8 ± 3.6	0.398	31.2 ± 4.1	31.8 ± 4.3	0.297
Time since delivery, mean months ± SD	11.3 ± 7.7	11.4 ± 7.6	11.0 ± 8.0	0.700	11.6 ± 7.6	10.9 ± 7.9	0.474
Race, n(%)							
White	255 (92.4)	192 (92.3)	63 (92.7)	0.927	161 (93.1)	94 (91.3)	0.585
Other^b^	21 (7.6)	16 (7.7)	5 (7.4)		12 (6.9)	9 (8.7)	
Marital status, n(%)^c^							
Married	255 (92.4)	189 (90.9)	66 (97.1)	0.117	162 (93.6)	93 (90.3)	0.310
Unmarried	21 (7.6)	19 (9.1)	2 (2.9)		11 (6.4)	10 (9.7)	
Education, n(%)							
Some college or less	52 (18.8)	39 (18.8)	13 (19.1)	0.524	34 (19.7)	18 (17.5)	0.416
Bachelor’s degree	119 (43.1)	94 (45.2)	25 (36.8)		79 (45.7)	40 (38.8)	
Master’s degree	83 (30.1)	58 (27.9)	25 (36.8)		49 (28.3)	34 (33.0)	
Professional or Doctorate degree	22 (7.9)	17 (8.2)	5 (7.4)		11 (6.4)	11 (10.7)	
Employment, n(%)							
Employed full time	128 (46.4)	99 (47.6)	29 (42.7)	0.307	82 (47.4)	46 (44.7)	0.253
Employed part time	41 (14.9)	27 (13.0)	14 (20.6)		21 (12.1)	20 (19.4)	
Homemaker	107 (38.8)	82 (39.4)	25 (36.8)		70 (40.5)	37 (35.9)	
Household income (*n* = 266), n(%)							
< $50,000	33 (12.4)	27 (13.4)	6 (9.2)	0.580	20 (12.1)	13 (12.9)	0.561
$50,000–$99,999	93 (35.0)	66 (32.8)	27 (41.5)		61 (37.0)	32 (31.7)	
$100,000–$149,999	82 (29.7)	63 (31.3)	19 (29.2)		46 (27.9)	36 (35.6)	
> $150,000	58 (21.8)	45 (22.4)	13 (20.0)		38 (23.0)	20 (19.8)	
Parity prior to twin pregnancy, n(%)							
Nulliparous	129 (46.7)	108 (51.9)	21 (30.9)	**0.003**	89 (51.5)	40 (38.8)	**0.042**
Multiparous	147 (53.3)	100 (48.1)	47 (69.1)		84 (48.6)	63 (61.2)	
Pre-pregnancy BMI Category, n(%)							
Underweight/normal weight^d^	142 (51.5)	114 (54.8)	28 (41.2)	**0.044**	93 (53.8)	49 (47.6)	0.556
Overweight	64 (23.2)	41 (19.7)	23 (33.8)		37 (21.4)	27 (26.2)	
Obese	70 (25.4)	53 (25.5)	17 (25.0)		43 (24.9)	27 (26.2)	
Use of assisted reproductive technologies, n(%)	105 (38.0)	77 (37.0)	28 (41.2)	0.540	64 (37.0)	41 (39.8)	0.642
Smoking in twin pregnancy, n(%)^c^	3 (1.1)	2 (1.0)	1 (1.5)	0.574	2 (1.2)	1 (1.0)	0.999
Alcohol use in twin pregnancy, n(%)^c^	7 (2.5)	5 (2.4)	2 (2.9)	0.683	4 (2.3)	3 (2.9)	0.715
MVPA, median minutes/day ± interquartile range^e,f^							
Before pregnancy (*n* = 262)	190.0 ± 255.0	200.0 ± 245.0	162.5 ± 197.5	0.089	205.0 ± 270.0	175.0 ± 225.0	0.568
First trimester (n = 262)	45.0 ± 175.0	70.0 ± 190.0	0.0 ± 87.5	**0.005**	30.0 ± 180.0	45.0 ± 125.0	0.460
Second trimester (*n* = 264)	0.0 ± 125.0	15.0 ± 140.0	0.0 ± 50.0	**0.015**	0.0 ± 140.0	0.0 ± 50.0	0.059
Third trimester (*n* = 261)	0.0 ± 0.0	0.0 ± 30.0	0.0 ± 0.0	0.053	0.0 ± 30.0	0.0 ± 0.0	0.298
Averages across trimesters (*n* = 273)	25.0 ± 113.3	33.3 ± 130.0	2.5 ± 50.0	**0.008**	33.3 ± 138.3	24.2 ± 63.3	0.243
Diet quality, mean score ± SD^f,g^							
Before pregnancy	3.3 ± 0.9	3.3 ± 0.8	3.4 ± 0.9	0.775	3.3 ± 0.8	3.3 ± 0.9	0.085
First trimester	3.1 ± 1.0	3.1 ± 1.0	2.9 ± 1.0	0.106	3.2 ± 0.9	2.9 ± 1.0	**0.012**
Second trimester	3.3 ± 0.8	3.3 ± 0.8	3.3 ± 0.8	0.894	3.4 ± 0.8	3.2 ± 0.8	0.086
Third trimester	3.3 ± 0.9	3.2 ± 0.9	3.4 ± 0.9	0.369	3.4 ± 0.9	3.1 ± 0.9	**0.019**
Averages across trimesters	3.2 ± 0.8	3.2 ± 0.8	3.2 ± 0.7	0.520	3.3 ± 0.7	3.1 ± 0.8	**0.003**
Twin pregnancy type, n(%)							
Diamniotic/dichorionic	221 (80.1)	164 (78.9)	57 (83.8)	0.372	135 (78.0)	86 (83.5)	0.272
Monoamniotic/dichorionic or monoamniotic/monochorionic	55 (19.9)	44 (21.2)	11 (16.2)		38 (22.0)	17 (16.5)	
Pregnancy complications, n(%)^h^	165 (59.8)	127 (61.1)	38 (55.9)	0.450	111 (64.2)	54 (52.4)	0.055
Gestational age at delivery, mean weeks ± SD	35.7 ± 2.1	35.6 ± 2.2	36.3 ± 1.8	**0.018**	35.5 ± 2.2	36.0 ± 1.9	0.052
Adequacy of GWG, n(%)^i^							
Below IOM guidelines	77 (27.9)	58 (27.9)	19 (27.9)	0.743	51 (29.5)	26 (25.2)	0.222
Within IOM guidelines	129 (46.7)	95 (45.7)	34 (50.0)		74 (42.8)	55 (53.4)	
Above IOM guidelines	70 (25.4)	55 (26.4)	15 (22.1)		48 (27.8)	22 (21.4)	
Twin A birth weight, n(%)^j^							
Very low or low birth weight	131 (47.5)	105 (50.5)	26 (38.2)	0.079	93 (53.8)	38 (36.9)	**0.007**
Normal or high birth weight	145 (52.5)	103 (49.5)	42 (61.8)		80 (46.2)	65 (63.1)	
Twin B birth weight, n(%)^j^							
Very low or low birth weight	127 (46.2)	101 (48.8)	26 (38.2)	0.130	88 (51.2)	39 (37.9)	**0.032**
Normal or high birth weight	148 (53.8)	106 (51.2)	42 (61.8)		84 (48.8)	64 (62.1)	

Abbreviations: *BMI* body mass index, *GWG* gestational weight gain, *IOM* Institute of Medicine, *MVPA* moderate to vigorous intensity physical activity, *SD* standard deviation

^a^*P*-value calculated using independent samples t-tests or chi-square tests unless otherwise indicated. Bolded values are statistically significant (p < 0.05)

^b^Black or African American (n = 6), American Indian or Alaska Native (*n* = 3), Asian (*n* = 7), Pacific Islander (*n* = 0), and Other (*n* = 5)

^c^*P*-value calculated using Fisher’s exact test

^d^Underweight and normal weight combined due to small sample of underweight women (*n* = 5)

^e^MVPA minutes/day assessed using the 2009 Behavioral Risk Factor Surveillance System physical activity questionnaire

^f^*P*-value calculated using Wilcoxon Mann Whitney test

^g^Diet quality score range 1–5, with higher scores indicating better diet quality

^h^Pregnancy complications include: gestational diabetes, high blood pressure or hypertension, preeclampsia, anemia, twin to twin transfusion syndrome, and hyperemesis gravidarum

^i^Using weekly rate of GWG across all trimesters

As seen in Table 2, 208 women or 74.5% of the study sample reported receiving provider advice on physical activity during their twin pregnancy. Of those who recalled provider advice on physical activity, 73.1% of women reported advice on physical activity type, 62.5% on physical activity intensity, and 45.7% on physical activity frequency/duration. When examining responses from open-ended questions, walking was the most commonly prescribed type of exercise (51.9%), followed by swimming (31.3%) and yoga (17.3%). Women who reported provider advice on physical activity intensity were most frequently encouraged to engage in light intensity activity (22.1%), light to moderate intensity activity (11.5%), or moderate intensity activity (13.0%). Of those who reported provider advice on physical activity frequency or duration, the most common recommendation was daily physical activity for 20–30 min (11.1%). However, it is important to note that relatively few women reported receiving quantitative physical activity recommendations from their provider. Nearly half of women also reported that provider advice on physical activity changed as their pregnancy progressed, with women often being told in later trimesters to listen to their body and don’t overdo it (19.2%), and exercise to comfort level (17.3%). Nearly 55% of women reported provider recommendations to restrict their physical activity levels, including strict or partial bed rest. The timing when physical activity restrictions were recommended varied: *“As soon as it was discovered that we were having twins, around 5.5 weeks, they advised me that I probably shouldn’t do any [physical activity].*” Another woman responded *“I could continue my normal routine until I was 20 weeks, then they [my provider] wanted me to just go for walks for exercise. I was on modified bed rest from 29-35 weeks.”*.

**Table 2:** Women’s report of provider advice on physical activity, *N*=208^a^

Provider Advice	N(%)^b^
Physical activity type	152 (73.1)
Walking	108 (51.9)
Swimming	65 (31.3)
Yoga	36 (17.3)
Low-impact activities	11 (5.3)
Jogging	10 (4.8)
Strength training	6 (2.9)
Work activities	5 (2.4)
Elliptical	4 (1.9)
Biking	3 (1.4)
Not sure if physical activity type was discussed	15 (7.2)
Missing	3 (1.4)
Physical activity intensity	130 (62.5)
Light	46 (22.1)
Light to moderate	24 (11.5)
Moderate	27 (13.0)
Heart rate references	8 (3.8)
Reduce intensity from pre-pregnancy	4 (1.9)
Not sure if physical activity intensity was discussed	19 (9.1)
Missing	2 (1.0)
Physical activity frequency and/or duration	95 (45.7)
2–3 days per week	3 (1.4)
3 days per week	6 (2.9)
3–5 days per week	8 (3.8)
Daily	18 (8.7)
0 min	6 (2.9)
Less than 20 min	3 (1.4)
20–30 min	23 (11.1)
Not sure if physical activity frequency/duration was discussed	25 (12.0)
Missing	3 (1.4)
Physical activity advice changed during pregnancy	99 (47.6)
Other emergent themes across categories	
Continue pre-pregnancy activities	48 (23.1)
Listen to body and don’t overdo it	40 (19.2)
Exercise to comfort level	36 (17.3)
Restrict physical activity levels	87 (41.8)
Bedrest prescribed	27 (13.0)

^a^Number of participants who reported receiving provider advice on physical activity during their twin pregnancy; used as denominator to calculate percentages

^b^Percentages may not sum to the total within a given domain as responses could be coded into multiple categories

As seen in Tables 3, 173 women or 62.7% of the study sample reported provider advice on nutrition during their twin pregnancy. Women commonly reported general recommendations to eat a well-balanced or healthy diet (38.7%). The most consistent recommendation was to increase protein intake (40.5%). One woman reported: “*Towards the end, the babies were measuring a little small on ultrasound so they [my provider] recommended more protein to help them gain weight.”* Women with gestational diabetes recalled specific advice to limit their carbohydrate and sugar intake. Of those who reported provider advice on nutrition, less than 12% recalled provider advice on fruit or vegetable consumption. Only 30% of women reported provider recommendations on caloric intake, with advice to increase by 300–1500 cal per day. Notably, few women reported provider advice on foods to avoid during pregnancy, such as deli meats or unpasteurized cheese (13.9%). Some women recalled receiving brochures (2.9%) or obtaining information on nutrition from classes (1.7%) or books (1.2%).

**Table 3:** Women’s report of provider advice on nutrition, *N* = 173^a^

Provider Advice	N (%)
Balanced/healthy diet	67 (38.7)
Macronutrients discussed	79 (45.7)
Protein	70 (40.5)
Carbohydrates	14 (8.1)
Fats	9 (5.2)
Fruits and/or vegetables encouraged	20 (11.6)
Micronutrients discussed	14 (8.1)
Water encouraged	15 (8.7)
Caloric recommendations	51 (29.5)
Increase intake	14 (8.1)
Increase by 300–500 cal	11 (6.4)
Increase by 600–1500 cal	8 (4.6)
1600–1800 total calories	4 (2.3)
2000–2500 total calories	8 (4.6)
2500–3500 total calories	14 (8.1)
Not sure if caloric recommendations were discussed	32 (18.5)
Specific diet types recommended	25 (14.5)
Gestational diabetes diet	23 (13.3)
Brewer diet	2 (1.2)
Foods to limit or avoid (e.g. deli meat)	24 (13.9)
Small meals, frequently	19 (11.0)
Continue pre-pregnancy diet	8 (4.6)
Don’t over-indulge	5 (2.9)
Alternate sources of dietary information	16 (9.2)
Dietician	6 (3.5)
Brochures	5 (2.9)
Class for gestational diabetes	3 (1.7)
Books	2 (1.2)

^a^Number of participants who reported receiving provider advice on nutrition during their twin pregnancy; used as denominator to calculate percentages

^b^Percentages may not sum to the total within a given domain as responses could be coded into multiple categories

Of the women who recalled provider advice on physical activity or nutrition during their twin pregnancy, 40.4% said they began the conversation on physical activity, compared to 20.8% for nutrition (see Table 4). Women most commonly reported receiving advice on physical activity and nutrition from an Ob/Gyn (86.5 and 77.5%, respectively), followed by a Maternal Fetal Medicine Specialist (26.0 and 24.9%, respectively). Approximately 70% of women who recalled provider advice on physical activity or nutrition were satisfied or very satisfied with the information received.

**Table 4:** Women’s report of aspects related to provider advice on physical activity and nutrition

Physical Activity, *N* = 208	N(%)^a^
Who started conversation about physical activity	
Participant	84 (40.4)
Provider	99 (47.6)
Not sure	25 (12.0)
Which health care provider discussed physical activity^b^	
Ob/Gyn	180 (86.5)
Maternal Fetal Medicine Specialist	54 (26.0)
Midwife	18 (8.7)
Nurse Practitioner	18 (8.7)
Nurse	14 (6.7)
Infertility Specialist	10 (4.8)
Dietician	6 (3.0)
Family Practitioner	2 (1.0)
Satisfaction with provider advice on physical activity	
Very dissatisfied	1 (0.5)
Dissatisfied	12 (5.8)
Neutral	49 (23.6)
Satisfied	92 (44.4)
Very Satisfied	54 (26.0)
Nutrition, *N* = 173	
Who started conversation about nutrition	
Participant	36 (20.8)
Provider	114 (65.9)
Not sure	23 (13.3)
Which health care provider discussed nutrition^b^	
Ob/Gyn	134 (77.5)
Maternal Fetal Medicine Specialist	43 (24.9)
Midwife	13 (7.5)
Nurse Practitioner	15 (8.7)
Nurse	14 (8.1)
Infertility Specialist	2 (1.2)
Dietician	25 (14.5)
Family Practitioner	3 (1.7)
Satisfaction with provider advice on nutrition	
Very dissatisfied	4 (2.3)
Dissatisfied	11 (6.4)
Neutral	35 (20.2)
Satisfied	82 (47.4)
Very Satisfied	41 (23.7)

^a^Percentages calculated from those who reported advice on physical activity (*N* = 208) or nutrition (*N* = 173)

^b^Participants were able to select more than one response

In exploratory analyses, we examined whether there was an association between who initiated the conversation on physical activity or nutrition (self vs. provider) with the corresponding lifestyle behavior before and during pregnancy as well as satisfaction with advice. As seen in Table 5, women who reported self-initiated conversations vs. provider-initiated conversations on physical activity had more median MVPA minutes/day (245.0 vs. 175.0) before their twin pregnancy, as well as in the first (142.5 vs. 30.0) and second trimesters (75.0 vs. 0.0) and across pregnancy trimesters (61.7 vs. 22.5) (*p* ≤ 0.01 for all). Conversely, women who reported provider-initiated conversations vs. self-initiated conversations on nutrition had higher mean diet quality scores in the second (3.5 vs. 3.2) and third trimesters (3.5 vs. 3.2), as well as across pregnancy trimesters (3.4 vs. 3.1) (*p* < 0.05 for all). Women who reported provider-initiated conversations vs. self-initiated conversations on physical activity and nutrition were more likely to report being satisfied with the advice received (physical activity: 76.8% vs. 60.7%, *p* = 0.051; nutrition: 83.3% vs. 38.9%, *p* < 0.001; data not shown).

**Table 5:** Bivariate associations between self- or provider-initiated conversations on physical activity and nutrition and corresponding behaviors

MVPA, median minutes/day ± IQR^a^	Self-initiated Conversation on Physical Activity *N* = 84	Provider-initiated Conversation on Physical Activity *N* = 99	*p*-value^b^
Before pregnancy	245.0 ± 250.0	175.0 ± 220.0	**0.003**
First trimester	142.5 ± 257.5	30.0 ± 130.0	**< 0.001**
Second trimester	75.0 ± 200.0	0.0 ± 102.5	**0.010**
Third trimester	0.0 ± 75.0	0.0 ± 30.0	0.259
Averages across trimesters	61.7 ± 183.3	22.5 ± 88.3	**0.002**
Diet quality, mean score ± SD^c^	Self-initiated Conversation on Nutrition *N* = 36	Provider-initiated Conversation on Nutrition *N* = 114	*p*-value^b^
Before pregnancy	3.4 ± 0.9	3.3 ± 0.9	0.456
First trimester	3.1 ± 0.8	3.3 ± 0.9	0.273
Second trimester	3.2 ± 0.8	3.5 ± 0.8	**0.021**
Third trimester	3.2 ± 1.0	3.5 ± 0.9	**0.044**
Averages across trimesters	3.1 ± 0.7	3.4 ± 0.7	**0.025**

Abbreviations: *MVPA* moderate to vigorous physical activity, *IQR* interquartile range, *SD* standard deviation

^a^MVPA minutes/day assessed using the 2009 Behavioral Risk Factor Surveillance System physical activity questionnaire

^b^*P*-value testing for differences in physical activity and diet quality by women’s report of who initiated these conversations using Wilcoxon-Mann-Whitney tests

^c^Diet quality score range 1–5, with higher scores indicating better diet quality

## Discussion

Approximately 75% of study participants reported provider advice on physical activity and 63% on nutrition during their twin pregnancy. These findings are largely consistent with other studies examining provider counseling on lifestyle behaviors in singleton pregnancies, where 63–65% of women reported provider advice on physical activity and 56–69% reported provider advice on nutrition [11, 22]. However, it appears women pregnant with twins are more likely to report counseling on physical activity compared to women pregnant with singletons. This may be due to the greater risk of adverse pregnancy outcomes observed among twin gestations [2, 3], which could lead providers to recommend physical activity restriction, or alternately, women pregnant with twins may be more likely to initiate conversations on physical activity with their providers. While many women reported provider advice on physical activity and nutrition, it is concerning that 25–37% did not receive or recall advice on these important lifestyle behaviors.

We found multiple differences between those who reported advice on physical activity and nutrition and those who did not. Women who reported provider advice on physical activity also reported higher levels of MVPA during pregnancy than those who did not report provider advice on physical activity. Similarly, women who reported provider advice on nutrition had better diet quality during pregnancy than women who did not report provider advice on nutrition. These findings could indicate that provider advice on physical activity and nutrition has a positive influence on women’s behaviors in pregnancy, which is further supported by the high percentage of women who reported being satisfied with the advice they received on these topics. In previous work in singleton pregnancies, we found that provider advice on physical activity and nutrition was associated with women’s intentions to meet the guidelines for physical activity and nutrition [11]. Our exploratory analyses support this explanation for nutrition behaviors (but not physical activity), as women who reported provider-initiated conversations on nutrition had an improvement in diet quality scores across pregnancy trimesters, and also had higher diet quality scores compared to women who reported self-initiated conversations on nutrition. However, we also found that women with higher activity levels before pregnancy were more likely to initiate conversations with their providers on physical activity; thus individuals with healthier lifestyle behaviors may be more likely to initiate and/or recall conversations on physical activity or nutrition compared to those with poorer health habits.

Interestingly, women who reported provider advice on physical activity delivered earlier than women who did not report provider advice on physical activity, and women who reported advice on nutrition were more likely to have very low or low birth weight babies compared to those reporting no nutrition advice. Potential explanations for these findings are that providers were restricting physical activity among women with early signs of preterm labor, and conversations on diet may have focused on encouraging appropriate caloric or protein intake if babies were measuring small or women were not gaining adequate weight.

When examining the content of physical activity conversations, few women reported provider advice consistent with the ACOG or HHS guidelines [4, 5]. Specifically, only 13% of women were advised to engage in moderate intensity activity, with more reporting advice to engage in light intensity activity, which is currently not part of the ACOG or HHS guidelines. Also, less than 3% of patients reported advice on strength training, which is recommended in the ACOG guidelines [4]. However, given the high percentage of women reporting one or more pregnancy complications (60%), advice to engage in light-intensity activity at a lower volume may have been warranted. Of concern, 42% of women were recommended to restrict their activity levels, with an additional 13% prescribed bed rest. In the United States and elsewhere, bed rest is commonly prescribed to treat preterm labor and other diagnoses indicating increased risk of preterm birth [23, 24]. However, there is limited evidence supporting the efficacy of activity restriction to decrease preterm birth, perinatal mortality, or low birthweight in women pregnant with singletons or twins [25, 26]. On the contrary, there is evidence that activity restriction during pregnancy may have detrimental effects, including increased risk of thromboembolic events [27], bone loss [28], weight loss and muscle weakness [29], lower birth weight babies [30], and adverse psychosocial outcomes such as depression and anxiety [31, 32]. Given the lack of evidence supporting the beneficial effects of activity restriction or bedrest on pregnancy outcomes in twin pregnancies, it is recommended that providers have an informed discussion with their pregnant patients, outlining the unknown benefits of bedrest and potential adverse consequences [25].

When examining the content of provider advice on nutrition, women largely reported being told to eat a healthy, balanced diet with emphasis on protein intake. This is appropriate advice given the importance of adequate protein intake for fetal growth in twin pregnancies. For example, Moore and colleagues found that the percent of energy from protein in early pregnancy had a positive association with neonatal weight [33]. However, it is concerning that only 8% of women recalled provider advice on micronutrients (i.e., vitamins and minerals), especially given that recommendations for iron, folate, calcium, magnesium, and zinc supplementation is greater for twin pregnancies than what is included in a typical prenatal vitamin [7]. It is important to note that we did not specifically ask women about advice on micronutrients, which may have led to underreporting. In addition, less than 30% of women reported provider advice on caloric intake during their twin pregnancy. Given the known risks of inadequate maternal weight gain in twin pregnancies, including an increased risk of preterm delivery [34–36] and small for gestational age infants [36–40], it is critical that providers counsel women on the importance of appropriate caloric intake to ensure optimal health outcomes for the mother and infants.

It is imperative that providers and medical students receive physical activity and nutrition training to accurately and effectively counsel their patients on healthy lifestyle behaviors. Currently, existing medical school programs report limited physical activity and nutrition training in their curriculum [14, 41]. However, there are examples of medical schools, like the University of South Carolina Greenville, who have made it a priority to integrate physical activity into all 4 years of their curriculum [42]. Other programs like the University of Michigan and the University of California San Francisco have also revised their curriculum in recent years to offer greater flexibility in teaching multidisciplinary topics, including nutrition and weight management [43]. Therefore, while there is increasing pressure on medical programs to teach a wide variety of preventative practices [44], a comprehensive curriculum that integrates physical activity and nutrition content into medical school appears possible. For existing providers, continuing education programs could be a venue through which to provide these additional training opportunities. It is critical that health care providers, including those who work with pregnant women, have knowledge of the current physical activity and nutrition guidelines and ability to counsel women on these topics or provide appropriate referrals.

This is one of the first studies to examine the prevalence and content of provider advice on physical activity and nutrition among women pregnant with twins, a population at high risk for adverse maternal and neonatal outcomes. However, there are several study limitations to acknowledge. First, women were asked to self-report provider advice on physical activity and nutrition up to 3 years postpartum, and accuracy of recall may decrease over time. Notably, we did not find that a longer postpartum period was associated with reported provider advice. In addition, the majority of participants were < 12 months postpartum (63%). Second, women’s report of provider advice was not verified by the providers themselves. Additional research is needed to assess health care provider recall of conversations on physical activity and nutrition in women pregnant with twins. Third, to minimize participant burden we asked about nutrition advice more broadly, rather than asking if providers discussed specific content areas within nutrition (such as micronutrient recommendations), which may have limited the depth of participant responses. Qualitative study designs would allow additional opportunities to probe for details related to provider advice on both nutrition and physical activity. Fourth, the physical activity survey used in this study has not been previously validated in pregnant populations; however, the survey has been validated in other adult populations [17–19]. Fifth, we did not assess whether participants experienced absolute or relative contraindications to exercise, which would provide additional contextual information that could explain provider advice to restrict activity. Finally, this study was limited to predominately white, highly educated women who were recruited from social media websites targeting mothers of multiples, which limits generalizability of study findings. It is possible that women in this study had access to higher quality prenatal care, where providers were more likely to discuss physical activity and nutrition, as compared to the general population. Alternately, women who were more interested in healthy lifestyle behaviors during pregnancy may have been more likely to take part in this study.

In conclusion, the majority of women report receiving physical activity and nutrition advice from their health care provider during their twin pregnancy. However, it is concerning that approximately one-third of women did not report provider advice on these important lifestyle behaviors, which are known to influence pregnancy outcomes. Advice on physical activity and nutrition was limited in detail, and many women recalled recommendations to limit physical activity, despite the lack of evidence showing a beneficial effect of activity restriction on pregnancy outcomes. More research is needed to better understand optimal physical activity and dietary patterns in women pregnant with twins to better inform provider counseling, and there is a clear need for providers to receive additional training in physical activity and nutrition.

## Data Availability

The datasets used and analyzed during the current study are available in the University of Iowa’s Institutional Repository [10.25820/mj2q-gj21].

## Abbreviations

ACOG: American College of Obstetricians and Gynecologists
BMI: Body mass index
BRFSS: Behavioral Risk Factor Surveillance System
HHS: United States Department of Health and Human Services
MVPA: Moderate to vigorous intensity physical activity
